# Protein Extraction from Mealworm (*Tenebrio molitor*): Effects of Euthanasia and Drying on Yield and Composition

**DOI:** 10.3390/foods15030585

**Published:** 2026-02-05

**Authors:** Geert R. Verheyen, Sarah Goossens, Sabine Van Miert

**Affiliations:** Centre of Expertise Sustainable Biomass and Chemistry, Thomas More University for Applied Sciences, Kleinhoefstraat 4, B-2440 Geel, Belgium; sarah.goossens@thomasmore.be (S.G.); sabine.vanmiert@thomasmore.be (S.V.M.)

**Keywords:** mealworm, alkaline extraction, protein yield, oil quality, proximate analysis

## Abstract

This study evaluated the effects of two euthanasia methods (blanching, blast freezing) and two drying methods (oven drying, freeze drying) on protein extractability, oil recovery, lipid oxidation, and nutritional composition of mealworm-derived full-fat flours, defatted flours, and protein concentrates. Protein extraction yields differed significantly among treatments (ANOVA, *p* < 0.001), except between blanched + freeze-dried and blast-frozen + oven-dried samples. Blast freezing resulted in higher protein extraction yields than blanching. Blast freezing markedly increased acid values (>40 mg KOH/g oil) relative to blanching (<5 mg KOH/g), while freeze drying increased peroxide values more than tenfold compared with oven drying. Ash contents ranged from 4 to 8% without a treatment effect. Defatting significantly reduced oil content and increased protein and chitin contents. Chitin was nearly absent in protein concentrates. In blast-frozen + oven-dried protein concentrates, the oil content was significantly up-concentrated compared with defatted samples. P, Mg, and K significantly increased in defatted samples, while protein extraction reduced Mg, K, and Ca but increased Na due to alkaline solubilization. Micromineral profiles were most affected in protein concentrates, with increases in Cu and Fe and minor decreases in Mn. Overall, euthanasia and drying methods influence yield and quality, highlighting the need for tailored, scalable processing strategies for mealworm-based food and feed applications.

## 1. Introduction

As the global population continues to grow to an estimated 9.7 billion by 2050 [1] and as traditional livestock production faces significant challenges like environmental degradation, resource depletion, and greenhouse gas emissions, the development of sustainable and efficient food sources is becoming more critical [2]. Consequently, there is growing interest in alternative protein sources that are produced sustainably and that are nutritionally healthy. Edible insects, like mealworms, are investigated as a promising alternative protein source due to their high nutritional value, low environmental footprint, and efficient feed conversion ratios [3,4] Additionally, they can be reared on organic waste, contributing to waste reduction and the establishment of a circular food system [5].

As reviewed in Rumpold and Schlüter [6] and more recently in Jantzen de Silva Lucas et al. [7], multiple studies emphasize the substantial protein amounts, essential fatty acids, and micronutrient profiles present in edible insects, underscoring their potential role in addressing global food security challenges. Rumpold and Schlüter reported the nutritional composition of 236 edible insects. Large variations in nutrient profile within and among species are observed, which depend on the development stage and nutritional intake of the insects but also on different analytical techniques used for analyses. However, the protein content (per dry weight) ranges from 40 to 60%, and the amino acid profile (including essential amino acids) meets WHO requirements, making insects an excellent protein source [6].

A lot of research has focused on the nutritional composition of the yellow mealworm (*Tenebrio molitor*). The existing literature demonstrates that the nutritional composition of mealworms can be modulated to some extent by the insect’s diet [8]. Our previous work indicates that protein content ranges from 40.7 to 52.3%, while fat content ranges from 18.6 to 35.5%, depending on the substrate fed to the mealworms. These data confirm the potential of mealworms as a novel and nutritionally valuable protein source [8].

In Europe, the production of insects for human consumption is regulated under the EU Novel Food legislation [9]. This regulatory framework mandates that any food not consumed to a significant extent within the EU prior to 15 May 1997 (which includes insects) must undergo a formal novel food authorization procedure. Based on the positive safety evaluations of the European Food Safety Authority (EFSA), multiple novel food dossiers on insects have been approved, including three dossiers on the use of mealworms [10,11,12]. This makes the lawful implementation of mealworms and their derived ingredients on the EU market possible [13].

Despite regulatory progress, consumer acceptance remains a significant barrier to widespread adoption of insects in food products, particularly in Western cultures where entomophagy is often met with disgust and strong aversion [14]. One key factor influencing this reluctance is the sensory perception of whole insects, which many consumers find unappealing [15]. Tuccillo and colleagues [16] investigated the willingness to eat insects in Italian consumers and observed that a low level of insect visibility was preferred, a result also observed in German [17] and American populations [18]. However, Ribeiro et al. reported a strong unwillingness of most participants in their survey to try bread supplemented with insect protein [19]. More research is thus needed on the motivators/demotivators that influence consumers and to establish interventions that improve acceptance toward insect ingredients [14].

Tasting sessions may be a way to decrease food neophobia, but insect integration into Western food culture will involve a transitional phase with minced or powdered insects incorporated into ready-to-eat preparations, as people are not ready to add insects to their diets in “whole form” [20]. In order to integrate insect ingredients in popular food formats like, e.g., protein bars, burgers, pasta, or bakery products, insects need to be processed [21,22,23]. Processing techniques can comprise killing, drying, grinding, defatting, and protein extraction, and each of these steps can be performed in a variety of ways using different technologies, including traditional preprocessing steps (e.g., freezing, blanching, oven drying, extractions and precipitations) and advanced technologies (e.g., enzymatic hydrolysis, ultrafiltration, ultrasound-assisted extraction, and high-pressure processing) aiming at improved efficiency and scalability [21,24].

Current insect-processing steps typically involve killing and drying steps followed by protein extraction by alkaline solubilization and acid precipitation [25]. These conventional processes for generating insect-derived ingredients may introduce losses in nutritional quality and overall yield.

A variety of killing strategies have been reported, including freezing, blanching, asphyxiation, desiccation, grinding, and high hydrostatic pressure [26,27]. Freezing and blanching seem to be the most employed methods of killing insects. Larouche et al. concluded that blanching is an appropriate killing method for black soldier fly larvae since it is rapid and effective, and it minimizes lipid oxidation, microbial contamination and colour alteration [26]. Similar results were obtained by Zhen et al. [27]. Numerous drying technologies, such as oven drying, fluidized bed drying, microwave drying, and freeze drying, have likewise been tested [21,28,29]. Khatun et al. evaluated the effects of freeze drying versus oven drying in house crickets and Jamaican field crickets and concluded that freeze drying showed higher colour quality, but oven drying had superior effects of nutritional and flavour characteristics [30]. No differences in nutritional content (proximate, mineral, fatty acid composition) were observed among oven-dried or freeze-dried (blanched) longhorn grasshoppers (*Ruspolia differens*), indicating that either drying method could be used to preserve nutrient properties [31]. Kröncke and colleagues investigated the effect of oven drying, freeze drying and vacuum drying on the nutrient quality of yellow mealworms [32]. Proximate composition and fatty acid profiles were similar among all treatments, but colour and volatile compound profiles were different, with rack oven drying causing pronounced darkening with low content of volatiles, and vacuum drying and freeze drying leading to enrichment of volatile Maillard reaction and lipid oxidation intermediates [32].

Further processing steps to produce protein ingredients can include a lipid removal step to produce defatted flour and a subsequent further protein extraction step [33]. Several publications have evaluated defatting and protein extraction of insects, showing the importance of the methods that are used (Soxhlet extraction, supercritical CO_2_ extraction, etc.), the solvents that are used (e.g., hexane, ethanol) as well as the conditions under which proteins are solubilized (e.g., water/insect ratio, pH of solubilization, temperature) and precipitated (e.g., pH, temperature) [34,35,36]. Zozo et al. evaluated the nutritional quality and structure of blanched and freeze-dried black soldier fly flours before and after defatting using hexane extraction [37]. They concluded that both types of flour can be used for food applications, but that defatted black soldier fly larvae have an improved proximate composition. Laroche and colleagues investigated the effect of six defatting methods on lipid extraction yield, fatty acid profiles and protein extraction yield in house crickets and mealworms. Insect meals were commercially obtained and were subjected to Soxhlet extraction using different solvents, three-phase partitioning, and supercritical CO_2_ extraction for defatting. Proteins were then isolated by solubilization in 0.25 M NaOH followed by isoelectric precipitation at pH 4.3–4.5. Lipid extraction yields ranged from 11.9 to 22.7% for house crickets and from 22.1 to 28.8% in mealworms. Ethanol increased lipid extraction yields irrespective of the insect meal or extraction method that was used. More variation was observed in protein extraction yields (range 12.4–38.9% for house cricket and 11.9–39.3% for mealworm) and protein purity, ranging from 70.1 to 78.5% in house cricket and from 72.7 to 75.4% in mealworms [38].

While the above-mentioned papers do study different killing or drying treatments in edible insect species, including the yellow mealworm, none of them directly compare the yields that are obtained or describe the effects on the composition of downstream protein ingredients (full-fat flour, defatted flour, protein concentrate) of all combinations of killing (blanching or blast freezing) and drying (oven drying or freeze drying). In addition, most published studies use different protocols and methods to extract the oil and proteins from insect materials. This makes it more difficult to extrapolate potential effects of killing and drying treatments on subsequent oil and protein yields and composition. For this article, we therefore selected blanching and freezing as the most common and operationally feasible killing approaches, and oven drying and freeze drying as the commonly used drying methods as preprocessing steps [39]. Using these preprocessing steps in mealworm larvae, we then set out to evaluate the effects on subsequent oil and protein extraction yields and on the nutritional and mineral composition of downstream protein ingredients (full-fat flour, defatted flour, and protein concentrate).

This article examines the extraction of mealworm proteins for food applications. The objective of this study was to identify a scalable protein extraction approach and to assess how different euthanasia (blanching vs. freezing) and drying (oven-drying vs. freeze-drying) methods influence both oil and protein yield and the resulting quality of these derived components.

## 2. Materials and Methods

### 2.1. Chemicals

All chemicals used in the procedures and analyses are analytical grade and were purchased from Merck (Darmstadt, Germany) and VWR Chemicals (Leuven, Belgium).

### 2.2. Mealworm Processing Strategy

**Figure 1:**
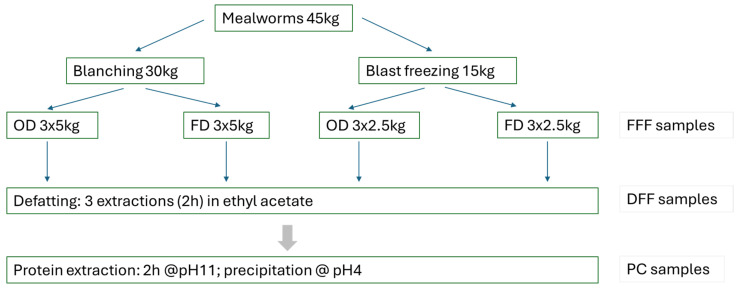
Scheme of the strategy used to extract proteins from mealworms. OD: oven dried, FD: freeze dried, FFF: full-fat flour, DFF: defatted flour, PC: protein concentrate.

The strategy used to extract the mealworm protein fractions is depicted in Figure 1.

A total of 45 kg of yellow mealworms was obtained from a commercial supplier (Nova-Res, Antwerp, Belgium). The insects were fasted for 24 h before euthanasia. Preliminary experiments in our lab have indicated that blanched mealworms have less extraction yield. Therefore, the mealworms were divided into a 30 kg batch destined for blanching and a 15 kg batch destined for blast freezing.

Euthanasia and drying: Blanching was done for 40 s in boiling water with a mealworm/water ratio of 1:10 in an indirect heating, electric boiling pan (MBM, Brescello, Italy). Blast freezing (Coldline W6TGN blast freezer, Torreglia, Italy) at −40 °C was performed for 2 h, and mealworms were subsequently stored at −20 °C.

After the euthanizing step, the mealworms were divided into three replicate batches for each of the subsequent processing/treatment steps.

Oven drying: mealworms were placed in 2–3 cm layers on perforated trays in a drying oven (Universal oven UF750, Memmert, Labconsult, Brussels, Belgium) at 65 °C. Oven drying was performed overnight (16 h) after which the trays were weighed. The drying process was continued until no further decrease in weight was observed, which could take up to 20 h.

Freeze drying: mealworms were placed in 2–3 cm layers on trays for freeze drying at −48 °C under 0.200 mbar using a Lyovapor L200 (Büchi, Breda, The Netherlands). The freeze-drying process was continued for 72 h, which in our experience corresponds to <10% moisture.

Defatting: Dried mealworms were ground and then defatted 3 times using ethyl acetate as solvent. In the first defatting step, a mealworm/solvent ratio of 1:3 was used. The extraction was performed during 2 h at room temperature with stirring at 400 rpm using an overhead stirrer (Heidolph Instruments, Schwabach, Germany). After 2 h, the mixture was placed over a filter (Qualitative filter paper, 454, particle retention 12–15 µm, VWR, Leuven, Belgium) to separate the solids from the oil/solvent mixture, and the mealworm powder was extracted again two times in a 1:2 mealworm/solvent ratio. After the final extraction, the defatted powder was left to dry overnight (at least 16 h) in a fume hood at room temperature. The solvent was evaporated and recovered using a rotavapor device (Rotavapor RS220; Büchi, Breda, The Netherlands), and the oil was retained for analysis.

Protein extraction: The defatted powder was placed in a 1:10 volume of demineralized water, and pH was adjusted to 11 with 33% NaOH. The mixture was incubated for 2 h with stirring at 400 rpm using an overhead stirrer (Heidolph Instruments, Schwabach, Germany). After this, the mass was centrifuged at 7000× *g* for 15 min at room temperature (Sorval Lynx 4000 centrifuge; Thermo Fisher Scientific, Geel, Belgium), and the supernatant was retained. The pH of the supernatant was lowered to 4 by adding HCl (37% solution) to allow the precipitation of proteins to occur, and the mass of the protein pellet was measured after centrifugation at 7000× *g* for 15 min at 8 °C.

### 2.3. Determination of Yields

Oil yield was calculated by dividing the resulting oil mass by the starting mass of the full-fat flour, expressed as percentage.

Protein yield was calculated by dividing the protein concentrate mass (corrected for % dry matter (%DM)) by the defatted flour mass (corrected for %DM), expressed as percentage.

Protein extraction yield was calculated by Formula (1):

(1) $$protein\;extraction\;yield\;(\%)=\;\frac{mass\;PC\;\times \;purity\;PC\;}{mass\;DFF\;\times \;purity\;DFF}$$

The masses and the purities of the protein concentrate (PC) and defatted flour (DFF) are corrected for the %DM.

### 2.4. Proximate Analyses

Dry matter content (DM): DM was determined by drying the sample in an oven at 105 °C for 24 h (Universal oven UF110, Memmert, Labconsult, Brussels, Belgium). DM percentage was calculated based on the weight loss of the sample.

Ash content: Crude ash content was determined from the weight loss by incineration in a muffle furnace (L9/11/SKM muffle furnace, Nabertherm, Lilienthal, Germany) at 550 °C for 4 h.

Crude fat: Crude fat content or ether extraction (EE) was performed with petroleum ether (BP 40–60 °C) using Soxhlet equipment. About 10 g of sample was placed in an extraction thimble and covered with ceramic wool to prevent sample loss. The sample was extracted overnight with 150 mL of petroleum ether. The extract was cooled down, and the organic solvent was removed and recovered via rotary evaporation (Rotavapor R-300; Büchi, Breda, The Netherlands) at 300 mbar and 50 °C. The crude lipid was cooled and weighed on an analytical balance.

Crude protein: Crude protein content (CP) was determined by analysing the nitrogen present in the samples using the Kjeldahl method [40]. Appropriate N to P factors were used to calculate the protein content. A factor of 5.33 was used for full-fat flour (FFF) samples and defatted flour (DFF) samples and of 5.6 for protein concentrates [41].

Chitin content: To determine the chitin content of the larvae, an adapted protocol was used based on analysis of crude fibre content following the Van Soest method [42]. A 1 g sample was placed in a dedicated fibre bag (CF bag, Gerhardt, Königswinter, Germany) and glass spacer. The fibre bag was filled with sample, defatted in petroleum ether (BP 40–60 °C) and air dried. The defatted sample was first extracted in an acidic solution of 0.13 M sulphuric acid for 30 min at boiling temperature. The extracted fibre bag with sample was rinsed three times with boiling, demineralized water and subsequently extracted in solution of 0.23 M potassium hydroxide for 2 h. The extracted fibre bag with the sample was again rinsed three times with boiling, demineralized water and dried overnight at 105 °C. The dried bag with fibre residue was weighed on an analytical balance and incinerated at 550 °C for 4 h in a muffle furnace. Ashes were weighed, and fibre content was calculated based on the weight loss from the incinerated sample.

Mineral profile: Samples were prepared as described by [43], and the mineral profile was determined using ICP-OES (Optima 4300™ DV ICP-OES, Perkin Elmer, MA, USA). The macro-elements Na, Ca, P, K, Mg and micro-elements Zn, Cu, Fe, Mn were determined.

### 2.5. Oil Quality Measures

Acid value: The acid value of the oil was determined by titration with KOH using an automated titrator (T5, IPAC, Mettler Toledo, Zaventem, Belgium) equipped with a DGi116 electrode. First, 0.5 g of oil was dissolved in 60 mL of an ethanol/diethylether mixture (1:1) and titrated with 0.01 M KOH in ethanol solution. A blank measurement was performed on the ethanol/diethylether solution without addition of oil. The equivalence point of the titration was determined with the LabX software (Version 12.0.0, Build 728).

The acid value was calculated using Formula (2):

(2) $$acid\;value\;(mg\;KOH/g\;oil)=\frac{{(V}_{KOH,\;sample}-V_{KOH,\;blank})\;\times C_{KOH\;}\times 56.11}{m_{sample}}$$

V_KOH, sample_ and V_KOH, blank_ are the titrated volumes of the sample and the blank, respectively; C_KOH_ is the concentration of the KOH solution that was used; m_sample_ is the mass of the sample used; 56.11 is the molecular weight of KOH.

Peroxide value: The peroxide value of the oil was determined by measuring the amount of iodine formed by the reaction of peroxides with iodide ions. The iodine was determined by performing a redox titration with sodium thiosulphate using an automated titrator equipped with a DMi140-SC-electrode (T5, IPAC, Mettler Toledo, Zaventem, Belgium). Two grams oil was added to 10 mL acetic acid/CHCl_3_ solution (3:2). Then, 500 µL of a saturated KI solution was added. The solution was titrated using a 0.01 M sodium thiosulphate solution. As a blank, a sample without oil was titrated. The equivalence point of the titration was determined using the LabX software (Version 12.0.0 Build 728).

The peroxide value is calculated using Formula (3):

(3) $$peroxide\;value\;\left(\frac{meq\;O_{2}}{kg}\right)=\frac{\left(V_{sample}-\;V_{blank}\right)\times \;C_{Na2S2O3}*1000}{m_{sample}}$$

V_sample_ and V_blank_ are the titrated volumes of the sample and the blank, respectively. C_Na2SO3_ is the concentration of the sodium thiosulphate solution that was used, and m_sample_ is the mass of the sample used.

### 2.6. Colour Measurements

Colour of the FFF, DFF and PC powders was measured using a colourimeter (CR-5, Konica Minolta, Chiyoda-ku, Japan). The lightness value, L*, ranging from 0 (dark) to 100 (light), was determined and reported.

### 2.7. Statistical Analyses

The data were analysed using the JMP Student Edition 18.2.0 software package from JMP Statistical Discovery LLC (Cary, NC, USA). Evaluation was done using one-way ANOVA at a statistical significance level of 0.05, with Tukey HSD as post hoc test for pairwise comparisons among groups.

## 3. Results

**Figure 2:**
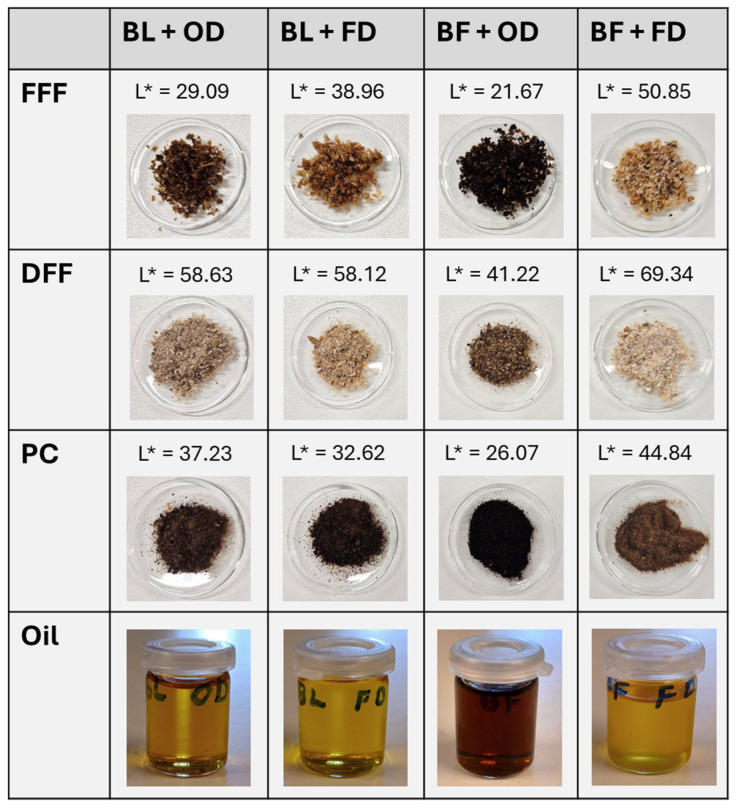
Images of the full-fat flour (FFF), defatted flour (DFF) and protein concentrate (PC) powders and the oil obtained after extraction for the four treatments: BL + OD: blanched and oven-dried; BL + FD: blanched and freeze-dried; BF + OD: blast-frozen and oven-dried; BF + FD: blast-frozen and freeze-dried. L* lightness value; higher values represent samples with lighter colour.

The protein powders and oils obtained after extraction had different appearances induced by the different treatments (Figure 2). The darkest powder and oil were observed after blast freezing and oven drying the mealworms. This is reflected in the L* values, which are lower (indicating a darker colour) than the values in the corresponding FFF, DFF and PC samples of the other treatments.

### 3.1. Oil Extraction and Quality

The first step of the process entails the solvent-based defatting of the ground full-fat flour (FFF). As shown in Figure 2, the BF + OD treatment produces an oil fraction with a markedly darker colouration, whereas all other treatments yield oils with a lighter, brown-yellowish appearance. Average oil yields are summarized in Table 1. ANOVA analysis indicates that there are significant differences among groups (*p* = 0.029). Cold extraction using ethyl acetate recovers approximately 25% oil from the initial FFF biomass. Oven-dried samples have slightly higher yields than freeze-dried samples; specifically, the blast-frozen and oven-dried sample yields statistically significantly more oil than the blast-frozen, freeze-dried treatment (Tukey post hoc test, *p* < 0.05).

Acid values (AVs) differ significantly among most treatments (ANOVA, *p* < 0.0001; Table 1), with the exception of the blast-frozen/oven-dried and blast-frozen/freeze-dried samples, which do not differ statistically. Blast freezing results in an approximately tenfold increase in free fatty acid content relative to blanched samples. Although the difference in AV between oven-dried and freeze-dried blanched samples is statistically significant, the magnitude of the difference is small. Peroxide values (PVs) also differ significantly among treatments (ANOVA, *p* < 0.0001; Table 1), with freeze drying consistently producing higher PVs than oven drying.

### 3.2. Protein Extraction

The resulting protein powders were dark brown, with the blast-frozen/oven-dried (BF + OD) samples exhibiting the darkest colouration. Protein yields and protein extraction yields for all treatments are presented in Table 2.

Both protein yield and protein extraction yield differ significantly among treatments (both ANOVA *p* < 0.001; Table 2). Tukey HSD indicates which pairwise comparisons are significantly different. Thermal treatments (blanching and oven drying) decrease the proportion of protein that precipitates at pH 4, whereas inclusion of a low-temperature step (blast freezing and freeze drying) increases the amount of recoverable protein. The highest extraction yield (30.31%) is obtained from the BF + FD treatment.

### 3.3. Proximate Composition of the Fractions

**Figure 3:**
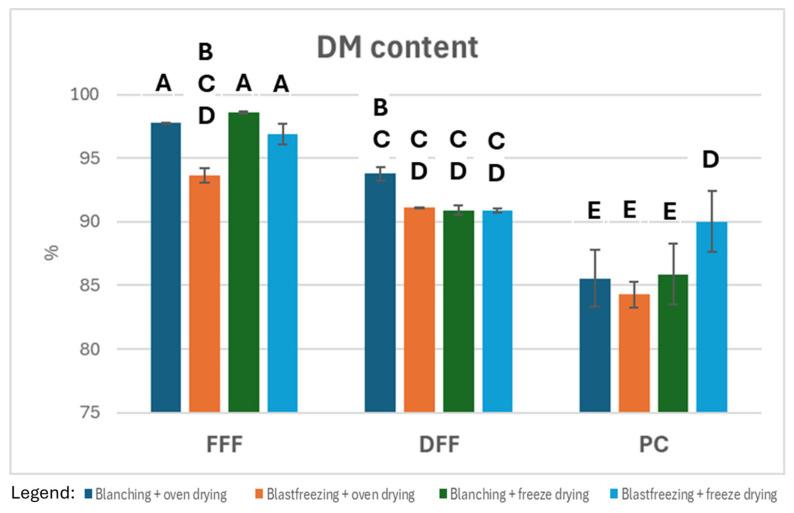
Dry matter content of mealworm fractions under the different treatments (N = 3/treatment). The bars represent the mean value (%) with standard deviation. Statistical significance among treatments is evaluated by Tukey HSD test (*p* < 0.05). Samples connected by a letter are not statistically significantly different.

Figure 3 summarizes the dry matter contents for the different treatments. ANOVA indicates that there are significant differences among the treatments (*p* < 0.0001). Full-fat flours (FFFs) retain minor moisture after drying (<6%). Defatted flours (DFFs) exhibit higher moisture contents (6–10%), which may partially represent residual ethyl acetate. Protein concentrates (PCs) contain up to 15% moisture.

**Figure 4:**
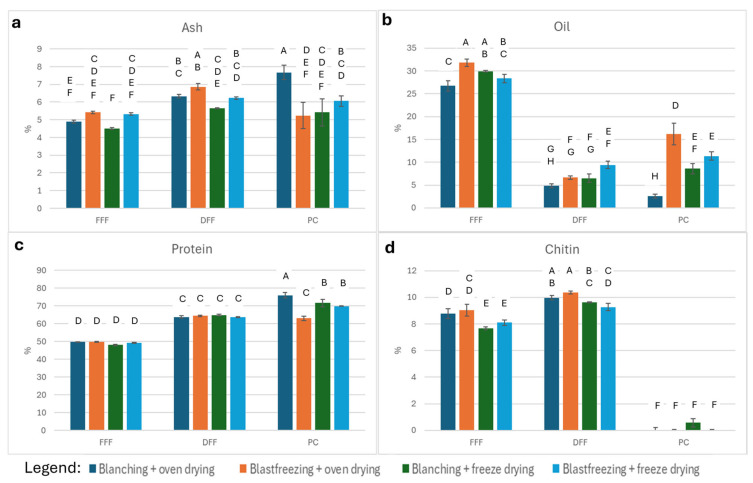
Proximate composition of the mealworm fractions under the different treatments (N = 3/treatment): (**a**): ash content; (**b**): oil content; (**c**): protein content; (**d**): chitin content. The bars represent the mean value (%) with standard deviation. Statistical significance among treatments is evaluated by Tukey HSD test (*p* < 0.05). Samples connected by a letter are not statistically significantly different.

Proximate compositions (crude ash, fat, protein, and chitin) are shown in Figure 4. All values are corrected for dry matter. Protein content of FFF and DFF were calculated using a N-to-protein (N-to-P) conversion factor of 5.33, whereas a factor of 5.6 was used for PC samples [41]. Statistical differences among treatments are indicated by letters: samples connected by a letter are not statistically significantly different.

Figure 4a presents the average ash content of the different treatments together with the results of pairwise comparisons of the average ash content among treatments. ANOVA demonstrates significant treatment effects on ash content (*p* < 0.0001). However, no consistent trend across treatment types is evident (Figure 4a).

Oil contents differ significantly among treatments (ANOVA *p* < 0.0001; Figure 4b). FFF samples contain, on average, 29.21% crude fat. Defatting reduces fat content to 6.85% in DFF samples. Three of the four PC samples exhibit an increased (compared with DFF samples) relative fat concentration, likely due to the concentration effect of removing other biomass components.

Protein contents also differ significantly among treatments (ANOVA *p* < 0.0001; Figure 4c). FFF samples contain 49.19% crude protein on average; this increases to 64.17% in DFF samples and 70.15% in PC samples. The highest protein purity (76.03%) occurs in the blanched and oven-dried treatment.

Chitin contents differ significantly among treatments (ANOVA *p* < 0.0001; Figure 4d). FFF contains an average of 8.39% chitin, increasing slightly to 9.81% in DFF samples. Chitin is almost entirely removed in PC samples.

### 3.4. Macrominerals

Average macromineral concentrations for each treatment are reported in Table 3, with statistical differences indicated by connecting letters.

Phosphorus (P) contents differ significantly among treatments (ANOVA *p* < 0.0001; Table 3). FFFs contain, on average, 925.8 mg/100 g DM, with no significant variation among FFF treatments (Table 3). In DFF samples, P increases to 1335.97 mg/100 g DM, again without significant differences among defatting treatments (Table 3). PC samples contain an average of 1046.89 mg/100 g DM. Blanched PC samples show significantly elevated P relative to FFF, whereas blast-frozen PC samples show lower P, with BF + OD significantly lower than FFF.

In FFFs, Mg is slightly lower in freeze-dried samples (238.1 mg/100 g DM) compared with oven-dried samples (254.1 mg/100 g DM), although the absolute differences are small. Defatting significantly increases Mg to an average of 323.6 mg/100 g DM across DFF treatments. In PC samples, Mg is nearly absent in blanched samples (12.96 mg/100 g DM) and entirely absent in blast-frozen samples, indicating almost complete removal during protein isolation (Table 3).

K contents do not differ among FFF treatments (average 1165.4 mg/100 g DM). Defatting significantly increases K to 1528.3 mg/100 g DM, without differences among DFF treatments. In PC samples, K decreases markedly to 404.7 mg/100 g DM, with the lowest values observed in the BF + FD treatment (Table 3).

Sodium (Na) contents do not differ significantly among FFF or DFF samples. Na increases significantly in PC samples due to NaOH addition during protein extraction, with the highest concentrations found in BF + OD samples (Table 3).

Ca differences among FFF, DFF, and PC samples are modest in magnitude. DFF samples have slightly higher Ca contents than FFF samples, but the differences are small. PC samples have significantly lower Ca contents than both FFF and DFF samples (Table 3).

### 3.5. Microminerals

**Figure 5:**
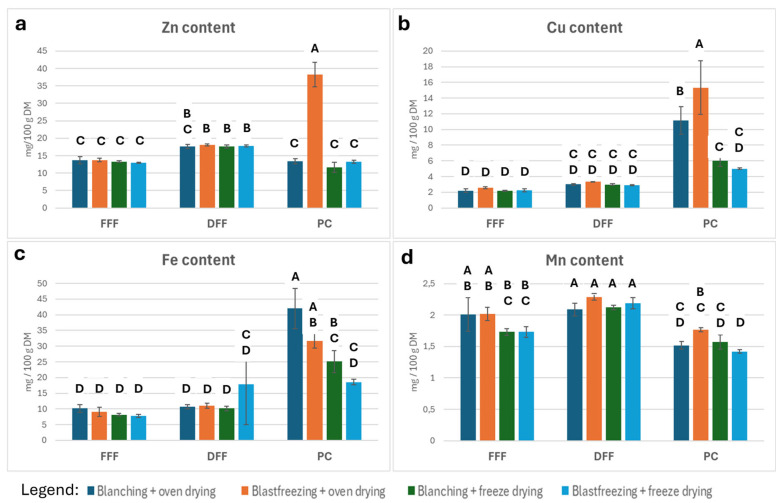
Micromineral content of the different treatments: (**a**) zinc (Zn); (**b**) copper (Cu); (**c**) iron (Fe); (**d**) manganese (Mn) (N = 3/treatment). The bars represent the mean value (%) with standard deviation. Statistical significance among treatments is evaluated by Tukey HSD test (*p* < 0.05). Samples connected by a letter are not statistically significantly different.

The micromineral profiles of the different treatments are shown in Figure 5, with corresponding Connecting Letter Reports indicating statistically significant differences among treatments.

Zinc (Zn): FFFs contain, on average, 13.4 mg Zn/100 g DM. Zn levels increase significantly in DFF samples (17.76 mg/100 g DM), except for blanched/oven-dried DFF. Most PC samples have Zn concentrations similar to FFF, except for BF + OD PC, which shows a substantial and significant increase (38.24 mg/100 g DM; Figure 5).

Copper (Cu): Cu contents do not differ significantly among FFF and DFF samples. In PC samples, Cu increases moderately (but not statistically significantly) in freeze-dried treatments (5.5 mg/100 g DM) and increases significantly in BF + OD (15.33 mg/100 g DM) and BL + OD (11.17 mg/100 g DM) treatments (Figure 5).

Iron (Fe): Fe contents are slightly higher in DFFs (12.4 mg/100 g DM) than in FFFs (8.79 mg/100 g DM), although this difference is not statistically significant. Fe increases significantly in oven-dried PC samples (35.86 mg/100 g DM) and in freeze-dried PCs (21.84 mg/100 g DM). The DFF BF + FD treatment also exhibits a comparatively elevated Fe level (17.82 mg/100 g DM; Figure 5).

Manganese (Mn): Mn contents are <3 mg/100 g DM across all samples. The highest Mn levels (>2 mg/100 g DM) occur in DFF samples and oven-dried FFF samples. Slightly lower values (~1.7 mg/100 g DM) appear in FFF freeze-dried samples and BF + OD PC samples. The lowest Mn levels (~1.5 mg/100 g DM) occur in blanched PC samples and in the BF + FD PC treatment (Figure 5).

## 4. Discussion

Improving consumer acceptance of edible insects requires preprocessing steps, including euthanasia, drying, and grinding, to produce a full-fat flour (FFF). Additional downstream processes, such as oil and protein extraction, yield defatted flour (DFF), insect oil, and protein concentrates (PCs). However, these operations can alter the nutritional and functional properties of insect-derived ingredients [44]. In this study, we examined how killing (blanching or blast freezing) and drying (oven or freeze drying) affect yields and quality of mealworm ingredients across subsequent fractionation steps.

In the literature, a variety of killing strategies have been documented, including freezing, blanching, asphyxiation, desiccation, grinding, and high hydrostatic pressure [26,27]. Numerous drying technologies, such as oven drying, fluidized bed drying, microwave drying, and freeze drying, have likewise been tested [21,28,29]. We selected blanching and freezing as the most common and operationally feasible killing approaches, and oven drying as a cost-effective method known to produce quality comparable to freeze drying in several applications [39].

*Mealworm oil yields and quality*

Defatting of the ground mealworm biomass yielded, on average, 25.3% oil across treatments. Oil recovery is influenced both by diet and extraction method. In earlier work, we recovered ~32% oil (dry weight basis) via Soxhlet extraction with ethyl acetate and 25.54% using the same cold extraction protocol applied here [45]. Although defatting is commonly included prior to protein extraction, many authors do not report corresponding oil yields. Laroche et al. [38] compared solvent-based Soxhlet extraction, supercritical CO_2_ (sCO_2_), and tri-phase partitioning (TPP), reporting lower yields for sCO_2_ (22.1%) and TPP (23.7%) relative to solvent extraction (24.3–28.8%), consistent with our values. In our study, oven-dried samples have slightly higher yields compared with freeze-dried samples, with the blast-frozen/oven-dried mealworms showing statistically significant higher yield than the blanched and freeze-dried condition. The blast-frozen and freeze-dried condition also has lower yield, but this is not significant. It could be speculated that the disruption of cellular structures by oven drying increases subsequent solvent accessibility [46].

Blast-frozen + oven-dried mealworm oil is very dark, indicating significant browning occurring under these processing conditions. It was expected that this browning would also occur in blast-frozen + freeze-dried samples, but this was not observed. Although we have not measured Maillard reaction markers, this could indicate that thermal Maillard reactions are occurring in blast-freezing-killed mealworm samples during oven drying but that this activity is suppressed during freeze drying.

Acid values (AVs) differ significantly among blanching and blast-freezing treatments. Blast freezing results in an approximately tenfold increase in free fatty acid content relative to blanched samples, indicating that lipase activity remains active when insects are killed at low temperature, which is a phenomenon widely described in the literature.

Peroxide values (PVs) also differ among treatments, with freeze-dried samples (>25 mEq/kg oil) consistently producing statistically significantly higher PVs than oven-dried samples (<2 mEq/kg oil), suggesting rancidity of freeze-dried oil samples. Unfortunately, we were unable to measure lipase activity or to perform additional oxidation assays (e.g., p-anisidine assay, TBARS assay) to provide further information on lipid stability. Interestingly, this pattern contrasts with Bogusz et al., where freeze-dried mealworm oil had lower peroxide values than oven-dried oil [46]. It is not clearly described by Bogusz et al. [46] how their samples were blanched. Perhaps differences in the processing methods resulted in the contradictory findings. Similar to our results, Lenaerts et al. also concluded that freeze drying induced higher peroxide values than thermal treatment [47].

Because high peroxide values were present even in blanched + freeze-dried samples—where enzymes would have been inactivated—oxidation in freeze-dried mealworms is unlikely to be enzyme-driven. It can be hypothesized that antioxidant substances would be produced during oven drying, resulting in lower peroxide values compared with freeze drying, where those substances are not formed. Another hypothesis could be that due to freeze drying, the insect material becomes more porous, making the oil more accessible to oxygen and increasing oxidation reactions [47,48]. Further research is needed to investigate these hypotheses.

*Mealworm protein yield*

In the literature, several strategies to extract insect proteins have been described, ranging from conventional aqueous, salt-based, and alkaline extraction methods to non-conventional strategies using enzymes or advanced technologies such as microwaves, ultrasound or pulsed electric fields [49]. Alkaline extraction remains the most widely used method and was applied here. After defatting, proteins were solubilized at high pH, and non-protein components such as chitin were removed. Proteins were subsequently precipitated near pH 4.

Extraction of mealworm protein under the conditions used in this study seems to be inefficient, resulting in limited amounts of protein concentrate formed, with protein extraction yields ranging from 6.3 up to 30.31% (Table 2). According to the literature, the exact conditions at which the protein solubilization is performed may have a large impact on the extraction yields. Zhao et al. reported an optimized protocol using 0.25 M NaOH solution to defatted worm ratio of 1:15, temperature of 40 °C, and 60 min extraction time. Using these extreme alkaline conditions, they obtained a protein yield of 65.1% [34]. Mintah et al. used a 0.25 M NaOH solution (25:1 NaOH to BSF ratio and temperature 50 °C) to extract proteins from black soldier fly larvae and reported an extraction rate of 64.44% [25]. Azagoh et al. were able to extract 59.9% and 26.4% proteins from fresh mealworm larvae and mealworm larvae meal (larvae that underwent blanching and an oil-pressing pretreatment) [35]. Although pH was kept at 10, the extraction was performed at 45 °C on 5 g of initial material in 200 mL pH adjusted demineralized water. In this Azagoh study, it was observed that the pretreatment steps (blanching and oil pressing) negatively influenced the protein extraction yield. Laroche et al. used a solubilization step with 0.25 M NaOH (1:15 insect:0.25 N NaOH ratio) and precipitation at pH 4.3–4.5 for the isolation of mealworm larvae proteins. They obtained protein extraction yields ranging from 31 up to 38.9%. The starting material used was a commercially obtained mealworm meal [38]. Similarly, Santhosh et al. extracted proteins from mealworms using different conditions, including a 1:10 ratio of defatted mealworm meal and demineralized water (pH 10) and a 1:15 ratio of defatted mealworm meal and 0.25 M NaOH, followed by precipitation at pH 4.5. The alkaline condition at pH 10 resulted in an extraction yield of 5.63%, while the extraction with 0.25 M NaOH gave 38.83% protein extraction yield [36].

The above literature overview indicates that there are several factors that influence the protein extraction yield. The extraction volumes that are used and the temperature at which extraction is performed will have an effect. However, temperature and volumes all have an economic impact, and therefore, we decided to perform extractions (at a kg scale) at room temperature and an insect:water ratio of 1:10. As illustrated in Santosh et al., the most important factor that maximizes protein extraction yield is the pH (0.25 M NaOH). High pH will increase the solubility of proteins due to the ionization of acidic and neutral amino acids, but it will impact the denaturation of proteins and the formation of lysinoalanine crosslinks, which negatively affects the nutritional quality and suitability of the proteins for consumption [50]. This is the main reason for us to limit the pH to 11 in our study.

Pretreatment strongly impacted protein extraction yield in our study. Blanched mealworm larvae yielded significantly less protein than blast-frozen larvae, and the highest yields were obtained from blast-frozen + freeze-dried samples. These results suggest that thermal exposure during killing and oven drying likely induces protein denaturation, thereby reducing solubility.

Although studies have examined the impact of killing or drying on nutrient composition and oxidative stability [32,46,51,52], to our knowledge, effects on protein extraction yields have not been reported.

*Effects of processing on the nutritional composition of mealworm products*

The proximate composition of mealworms can vary significantly depending on the source from where they are derived and the substrate that they have been cultivated upon [53]. The proximate compositions of the full-fat flours obtained in this study were consistent with our earlier reports [8,53], including ash (range 4.5–7.67 g/100 DM), chitin (7.67–9.03 g/100 g DM), crude oil (range 26.73–31.81 g/100 g DM), and crude protein (range 47.99–49.9 g/100 g DM).

The ash content of defatted samples was slightly higher than that of the full-fat flours and the protein concentrates, but the differences were low. There were no treatment-specific patterns. Chitin levels were marginally higher in oven-dried than freeze-dried samples. Chitin was nearly absent from PCs, as expected.

There were statistically significant differences in oil content of the full-fat flours, but the differences cannot be linked to a specific killing or drying treatment. Defatting reduced the oil content in the defatted flours as intended, but in three out of four treatments, there was an up-concentration in the oil content in the final protein concentrate. Only in the blanched + oven-dried samples was there a further reduction in oil content. This observed up-concentration of the oil in protein concentrates may be undesirable, as the protein concentrate becomes more energy dense and decreases in nutritional value [37]. Moreover, the remaining oil may contribute to shorter shelf life due to oxidative rancidity [54]. Protein concentrations increased after defatting (~64 g/100 g DM) and increased further in PCs (highest: 76.03 g/100 g DM for blanched/oven-dried samples), inversely proportional to residual oil. In general, protein concentrates and isolates are derived from different sources like legumes, cereals, and insects, from which other constituents like carbohydrates, fats and minerals are extracted. Protein concentrates typically contain 35–80% of protein, while protein isolates have more than 90% [55]. The defatting and subsequent protein-extraction steps have a significant improvement in protein content, although a lot of protein is lost during the extraction procedure.

To our knowledge, no other studies have reported the effect of different combinations of killing and drying steps on the extraction of proteins in mealworms. Still, this information is relevant, as the different preprocessing steps clearly can influence the subsequent yield of the protein concentrates. The lack of treatment-related effects on the proximate composition of downstream protein ingredients is a positive result; however, effects on nutritional and techno-functional quality are still possible. Vlahova-Vangelova et al. reported higher protein and carbohydrate contents but lower oil contents of microwave-treated larvae compared with oven- or freeze-dried samples [52]. Some investigators have reported a lower protein composition of mealworms that have been freeze dried compared with other drying methods [46,51], but others report no such effects [47,56].

Overall, our results indicate that defatting, rather than killing or drying conditions, primarily determines protein concentrate purity.

*Effects of processing on the mineral composition of mealworm products*

Macromineral contents (P, Mg, Na, Ca and K) in the full-fat flour mealworm samples are consistent with the values reported by Noyens et al. [53].

Processing mealworms has effects on the mineral composition of the downstream protein products.

Defatting increased P, Mg, and K compared with the FFFs, but differences among treatments were negligible. PCs showed markedly reduced Mg and K contents, with Mg being undetectable in blast-frozen PCs. Potassium in PCs was less than half of the amounts present in FFFs.

Phosphorus behaved differently: blast-frozen PCs contained significantly less P than DFFs or FFFs, whereas blanched PCs contained more P than FFFs, suggesting that killing method influences P retention during downstream processing. Calcium levels were similar across FFFs and DFFs but significantly lower in PCs, independent of treatment. Sodium was unchanged in FFFs and DFFs but increased sharply in PCs because of NaOH addition during solubilization. For Na, there are no differences in content between any of the full-fat and defatted flours, but Na content is strongly increased in the protein concentrates, which is due to the addition of NaOH during solubilization.

The situation is slightly more complicated in the micromineral contents, as the patterns were more variable. Zinc increased in most DFF samples relative to FFFs and PCs, with an unexplained strong increase in the blast-frozen + oven-dried PC. Copper and iron were similar in FFFs and DFFs but elevated in PCs, particularly in blanched treatments. Manganese showed slight decreases in PCs relative to FFFs and DFFs, though absolute differences were small.

*Implications for food products*

Mealworm larvae are mainly investigated for their potential use in food and feed products. For these purposes, processing is required for several reasons, including compliance with legislation, consumer health protection and acceptance, improved processability, and the removal of undesirable and/or anti-nutritional factors. The processing steps evaluated in this study highlight several implications for the nutritional quality of mealworm-derived ingredients intended for food or feed use.

The nutritional composition of the full-fat flours closely aligns with our previously published findings [8,53]. Although a few statistically significant differences in nutritional and mineral composition were observed across treatments, these variations cannot be directly attributed to the processing steps, and the absolute differences remain small. On a dry-matter basis, mealworms meet the EU nutritional claims for “high protein” and “high fibre” (Annex of Regulation (EC) No. 1924/2006) [57]. Although not assessed here, our earlier work [45] indicates that mealworm oil complies with the claim “low in saturated fat.” Comparison of the mineral data obtained in this study with Regulation (EU) No. 1169/2011 [58] and the Annex to Directive 90/496/EEC [59] shows that yellow mealworms qualify as “high” in P, Mg, K, Zn, Cu, Fe, and Mn, as their levels per 100 g dry weight exceed 15% of the respective nutrient reference values. Only calcium levels are insufficient, consistent with previous reports. Sodium levels are comparable to earlier data [53].

As expected, defatted flours contained reduced oil levels and higher protein concentrations, with ash and chitin contents slightly increased. On a dry-matter basis, these flours also meet the EU nutritional claims for “high protein” and “high fibre,” but do not meet the “low fat” claim (threshold < 3 g/100 g). All mineral concentrations increased relative to the full-fat flours. From a nutritional perspective, defatted flours present a more favourable profile due to the reduction in oil content. No substantial treatment effects were observed on the defatting process.

Protein concentrates exhibited a modest increase in protein content of approximately 5–10%. For most treatments, oil levels increased slightly, and crude ash remained comparable to full-fat flour levels. Chitin was almost entirely removed, which may enhance availability of other nutrients or enhance protein digestibility and might improve textural and sensory properties in human food and/or feed applications [60]. Mineral composition was more strongly affected: Mg, K, and Ca levels were markedly reduced in protein concentrates, and P decreased in the blast-frozen samples. Sodium content increased in all protein concentrates due to NaOH addition during the solubilization step, while Fe and Cu levels also increased. These shifts indicate that mineral profiles change substantially during protein concentration, with mixed implications for nutritional quality.

The extraction process—solubilization at pH 11 followed by precipitation at pH 4—had a major impact on protein extraction yield. A key observation is that blanching and oven drying reduced extraction efficiency, likely due to heat-induced protein denaturation and aggregation. Higher pH values can improve extraction yields, as reported in several studies [36], but may also promote the formation of lysinoalanine crosslinks, which negatively affect protein nutritional quality [50]. Therefore, we limited the extraction pH to a maximum of 11, although some lysinoalanine formation cannot be excluded. Combining blast freezing with freeze drying resulted in the highest extraction yields and likely preserved native protein structure, minimizing denaturation. This approach may offer economic advantages; however, a heat treatment step remains necessary at some stage to reduce microbial loads when insect-derived ingredients are intended for food or feed applications.

## 5. Conclusions

This study demonstrates that the preprocessing steps applied to mealworm (*Tenebrio molitor*) larvae—specifically the methods used for killing and drying—significantly influence the yields, composition, and quality of oil and other downstream fractions like defatted flour and protein concentrates. Blast freezing preserved protein extractability more effectively than blanching, and freeze drying after blast freezing produced the highest protein extraction yields. In contrast, blanching and oven drying may increase protein denaturation, resulting in reduced solubility, leading to decreased yield. The impact of preprocessing on protein yield and protein properties may have repercussions for industrial implementation where yield and quality (e.g., for making hybrid meat products) is important. Food companies need to evaluate if it is worthwhile to produce protein concentrates or to use, for example, defatted flours.

Blast freezing introduced high acid values compared with blanched mealworms. Freeze drying produced substantially higher peroxide values than oven drying, indicating greater oxidative susceptibility, even when larvae were blanched prior to drying. These observations raise the hypothesis that oxidation in freeze-dried samples may be driven by non-enzymatic mechanisms.

Proximate and mineral compositions of full-fat and defatted flours were broadly comparable across treatments, with defatting emerging as the primary determinant of protein concentrate purity. Processing altered some mineral levels, particularly Mg, K, P, Cu and Fe. Some of these effects may be influenced by the preprocessing treatment.

Future research recommendations: The choice of the killing and drying method has important implications for the nutritional quality of mealworm-derived ingredients. Future research efforts should focus on understanding the mechanisms of how these processing steps influence oil and protein oxidation, protein solubility, mineral retention, etc. Additional research can focus on digestibility of the protein ingredients, on the bio-availability of the minerals, on sensory aspects of food products that are made using mealworm protein ingredients, and on whether these parameters are influenced by different preprocessing steps. These types of studies will support the development of optimized, scalable processing strategies for insect-based food applications.

## Figures and Tables

**Table 1:** Average oil yields (%) and quality measures (with standard deviation; N = 3). AV = acid value; PV = peroxide value.

Treatment	Yield (%)	AV (mgKOH/g oil)	PV (mEq/kg oil)
Blanched + oven dried	25.88 ± 0.98 A, B	1.87 ± 0.13 B	1.84 ± 0.43 A
Blanched + freeze dried	24.20 ± 0.30 A	4.18 ± 0.25 B	25.78 ± 3.62 B
Blast frozen + oven dried	26.27 ± 0.30 B	43.37 ± 4.28 A	0.22 ± 0.07 A
Blast frozen + freeze dried	24.91 ± 0.54 A, B	44.36 ± 3.06 A	48.35 ± 4.22 C

Statistically significantly different values (Tukey HSD post hoc test for pairwise comparisons) among treatments for yield, AV, or PV are indicated by a different letter.

**Table 2:** Protein yield (%) and protein extraction yield (%) for each treatment.

	BL + OD	BL + FD	BF + OD	BF + FD
Protein yield (%)	5.80 ± 0.17 D	9.18 ± 1.84 C	13.24 ± 1.37 B	27.89 ± 1.27 A
Protein extraction yield (%)	6.30 ± 0.16 C	9.55 ± 1.69 B	11.99 ± 1.43 B	30.31 ± 1.12 A

Protein yield and protein extraction yield are corrected for %DW. Statistically significantly different values (Tukey HSD post hoc test for pairwise comparisons) among treatments for protein yield or protein extraction yield are indicated by a different letter.

**Table 3:** Macromineral (P, Mg, K, Na, Ca) composition (average and standard deviation (SD)) of the different fractions. Statistically significant differences are evaluated with Tukey HSD test. Values connected by a similar letter are statistically not significantly different.

Treatment	P (mg/100 g DM)	Mg (mg/100 g DM)	K (mg/100 g DM)	Na (mg/100 g DM)	Ca (mg/100 g DM)
	Mean ± SD	Mean ± SD	Mean ± SD	Mean ± SD	Mean ± SD
FFF BL + OD	918.32 ± 39.4 C	255.8 ± 6.6 B	1174.0 ± 49.6 B	183.3 ± 5.8 D	59.0 ± 1.5 A, B, C
FFF BF + OD	940.3 ± 33.7 C	252.3 ± 6.7 B, C	1203.1 ± 39.8 B	176.7 ± 5.8 D	51.5 ± 1.9 A, B, C
FFF BL + FD	908.8 ± 23.9 C	238.6 ± 6.3 B, C	1126.2 ± 29.7 B	193.3 ± 5.8 D	50.0 ± 0.6 A, B, C
FFF BF +FD	935.8 ± 16.4 C	237.7 ± 2.9 C	1158.2 ± 16.3 B	170.0 ± 17.3 D	46.2 ± 4.3 C
DFF BL + OD	1306.6 ± 63.7 B	318.4 ± 6.2 A	1487.9 ± 14.7 A	203.3 ± 5.8 D	63.0 ± 1.4 A, B
DFF BF + OD	1353.4 ± 20.5 A, B	330.3 ± 11.3 A	1550.7 ± 23.6 A	161.7 ± 58.0 D	48.8 ± 16.6 B, C
DFF BL + FD	1334.1 ± 12.2 B	320.5 ± 8.1 A	1512.1 ± 43.8 A	236.7 ± 5.8 D	64.8 ± 1.3 A
DFF BF +FD	1349.7 ± 21.8 A, B	325.1 ± 2.6 A	1562.5 ± 15.0 A	226.7 ± 5.8 D	57.6 ± 1.4 A, B, C
PC BL + OD	1483.4 ± 9.9 A	6.0 ± 1.3 D, E	411.3 ± 26.1 C, D	533.3 ± 63.5 C	11.3 ± 0.6 D
PC BF + OD	630.1 ± 47.4 D	0.0 ± 0.0 E	426.8 ± 66.8 C, D	1333.3 ± 251.7 A	14.9 ± 1.1 D
PC BL + FD	1219.7 ± 131.5 B	20.0 ± 6.1 D	445.2 ± 12.1 C	696.7 ± 40.4 B, C	17.7 ± 0.8 D
PC BF +FD	854.4 ± 16.0 C	0.0 ± 0.0 E	335.4 ± 33.6 D	823.3 ± 161.7 B	16.5 ± 2.9 D

## Data Availability

The original contributions presented in this study are included in the article. Further inquiries can be directed to the corresponding author.

## Abbreviations

The following abbreviations are used in this manuscript:

FFF	Full-fat flour
DFF	Defatted flour
PC	Protein concentrate
BL	Blanching
BF	Blast freezing
OD	Oven drying
FD	Freeze drying
